# A multitransmit external body array combined with a ^1^H and ^31^P endorectal coil to enable a multiparametric and multimetabolic MRI examination of the prostate at 7T

**DOI:** 10.1002/mp.13696

**Published:** 2019-08-01

**Authors:** Bart W. J. Philips, Mark J. van Uden, Stefan H. G. Rietsch, Stephan Orzada, Tom W. J. Scheenen

**Affiliations:** ^1^ Department of Radiology and Nuclear Medicine (766) Radboud university medical center P.O. Box 9101 Nijmegen The Netherlands; ^2^ Erwin L Hahn Institute for Magnetic Resonance Imaging, UNESCO World Cultural Heritage Zollverein, Kokereiallee 7, Building C84 D‐45141 Essen Germany; ^3^ High Field and Hybrid MR Imaging University Hospital Essen D‐45147 Essen Germany

**Keywords:** 1H, 31P, endorectal coil, prostate cancer, spectroscopy

## Abstract

**Purpose:**

*In vivo* ^1^H and ^31^P magnetic resonance spectroscopic imaging (MRSI) provide complementary information on the biology of prostate cancer. In this work we demonstrate the feasibility of performing multiparametric imaging (mpMRI) and ^1^H and ^31^P spectroscopic imaging of the prostate using a ^31^P and ^1^H endorectal radiofrequency coil (ERC) in combination with a multitransmit body array at 7 Tesla (T).

**Methods:**

An ERC with a ^31^P transceiver loop coil and ^1^H receive (Rx) asymmetric microstrip (^31^P/^1^H ERC) was designed, constructed and tested in combination with an external 8‐channel ^1^H transceiver body array coil (8CH). Electromagnetic field simulations and measurements and *in vivo* temperature measurements of the ERC were performed for safety validation. In addition, the signal‐to‐noise (SNR) benefit of the ^1^H microstrip with respect to the 8CH was evaluated. Finally, the feasibility of the setup was tested in one volunteer and three patients with prostate cancer by performing T_2_‐weighted and diffusion‐weighted imaging in combination with ^1^H and ^31^P spectroscopic imaging.

**Results:**

Electromagnetic field simulations of the ^31^P loop coil showed no differences in the E‐ and B‐fields of the ^31^P/^1^H ERC compared with a previously safety validated ERC without ^1^H microstrip. The hotspot of the specific absorption rate (SAR) at the feed point of the ^31^P/^1^H ERC loop coil was 9.42 W/kg when transmitting on ^31^P at 1 W. Additional *in vivo* measurements showed a maximum temperature increase at the SAR hotspot of 0.7°C over 6 min on ^31^P at 1.9 W transmit (Tx) power, indicating safe maximum power levels. When transmitting with the external ^1^H body array at 40W for 2:30 min, the temperature increase around the ERC was < 0.3°C. Up to 3.5 cm into the prostate the ^1^H microstrip of the ERC provided higher SNR than the 8CH. The total coil combination allowed acquisition of an mpMRI protocol and the assessment of ^31^P and ^1^H metabolites of the prostate in all test subjects.

**Conclusion:**

We developed a setup with a ^31^P transceiver and ^1^H Rx endorectal coil in combination with an 8‐channel transceiver external body array coil and demonstrated its safety and feasibility for obtaining multiparametric imaging and ^1^H and ^31^P MRSI at 7T in patients with prostate cancer within one MR examination.

## 1 Introduction

Proton magnetic resonance spectroscopic imaging (MRSI) is used to study the *in vivo* metabolic profile of the prostate by detecting and localizing the levels of citrate, choline, creatine and polyamines.^1^, ^2^, ^3^, ^4^ An increase in the ratio of choline over citrate, which is partly attributed to increased levels of choline, has been described as a valuable biomarker to discriminate prostate cancer from healthy prostate tissue.^5^ It indicates prostate cancer specific changes in the phospholipid metabolism which plays an important role in cell membrane generation and degradation.^5^, ^6^, ^7^, ^8^ This phospholipid metabolism can be studied in more detail when using ^31^P‐MRSI to assess the levels of the phosphomonoesters (PMEs): phosphocholine (PC) and phosphoethanolamine (PE), and the phosphodiesters (PDEs): glycerophosphocholine (GPC) and glycerophosphoethanolamine (GPE).^9^, ^10^, ^11^ Parts of the PME and PDE signals, together with resonances of free choline, free ethanolamine, taurine, and myo‐inositol, comprise the total choline (tCho) peak at 3.2 ppm in ^1^H spectroscopy.^11^ Since ^31^P and ^1^H spectroscopy of the prostate provide complementary information on tumor biology, it would be of high interest to obtain both within one measurement session. Ideally, this would even be combined with T_2_‐weighted (T_2_W) and diffusion‐weighted imaging (DWI) of the prostate, as these are paramount in accurate localization and characterization of potential prostate cancer lesions.

The combination of ^1^H and ^31^P spectroscopy and ^1^H imaging requires a radiofrequency (RF) coil setup with transmit (Tx) and receive (Rx) capabilities on both nuclei,^12^ preferably one in which no coils need to be exchanged during the examination.

Because of the lower intrinsic magnetic resonance (MR) sensitivity of ^31^P compared to ^1^H and the low concentration of ^31^P metabolites in the human body, ^31^P spectroscopy of the prostate is challenging. ^31^P MRSI should preferably be performed at 7 Tesla with an endorectal radiofrequency coil (ERC) to optimize signal‐to‐noise (SNR) and to increase spectral resolution. However, for T_2_W imaging and DWI of the prostate at 7T, only using an endorectal coil is inadequate. Transmitting radiofrequency pulses with a small local surface coil results in substantial transition bands in T_2_W and DWI images due to its severely inhomogeneous transmit profile.^12^, ^13^ This can be solved by using a multichannel external body array in combination with B_1_^+^ shimming to provide a homogeneous transmit field.^13^, ^14^ Since an endorectal coil is needed for ^31^P, this ERC can also be equipped with ^1^H receive capabilities to enhance ^1^H imaging and spectroscopy of the prostate, increasing SNR for ^1^H spectroscopy and spatial resolution for T_2_W imaging and DWI. Moreover, the ^1^H transmit capabilities of the external body array allow the use of the nuclear Overhauser effect for an extra increase in ^31^P SNR.^15^

Therefore, we introduce and evaluate a coil setup with a ^31^P Tx/Rx – ^1^H Rx ERC in combination with an 8‐channel external multitransmit ^1^H array to enable multiparametric imaging and ^1^H and ^31^P spectroscopic imaging of the prostate at 7T. We perform electromagnetic field simulations, phantom measurements and *in vivo* temperature measurements to validate the safety of the setup. In addition, we evaluate its SNR performance and demonstrate the feasibility of the complete setup in three patients and a volunteer.

## 2 Methods

### 2.1 Subjects

One healthy volunteer (age 40 yr, weight 83 kg) and three patients (age: 60, 63, and 67 yr, weight: 97, 83, and 88 kg) with histopathologically proven prostate cancer were measured using the setup described below. An intramuscular injection of butylscopolamine bromide (Buscopan, Boehringer‐Ingelheim, Ingelheim, Germany) was used to suppress peristalsis. For all volunteer and patient measurements, informed consent was signed and the study was approved by the institutional review board.

### 2.2 Hardware

All MR measurements were performed on a 7‐Tesla whole‐body MRI system (MAGNETOM, Siemens Healthcare, Erlangen, Germany). Specific absorption rate (SAR) monitoring of the ^1^H and ^31^P measurements was performed with a custom build SAR supervision system, which measured the time‐averaged input power over 10 s and over 6 min. This input power was not to exceed the safety levels based on local SAR restrictions, otherwise the safety system would stop the measurement.

#### 2.2.1 Combined ^31^P Tx/Rx and ^1^H Rx endorectal coil ( ^31^P/ ^1^H ERC)

**Figure 1:**
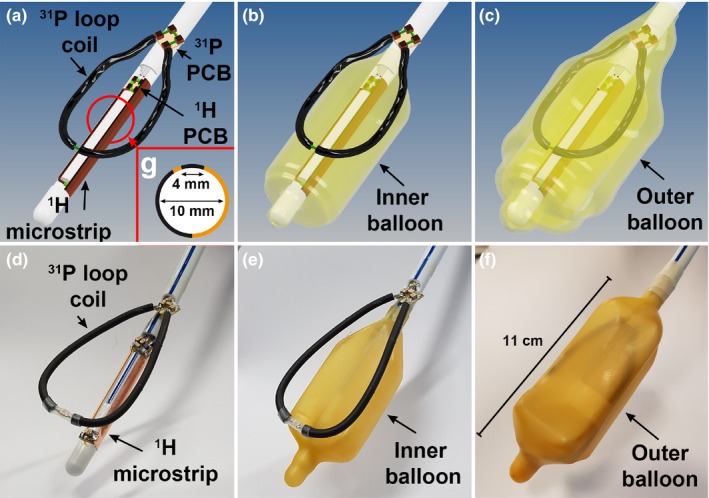
3D drawings of the endorectal coil without any balloon (a), with the inner balloon (b) and with the inner and outer balloon present (c). The ^31^P Tx/Rx loop is positioned between the balloons while the ^1^H Rx elements is attached to the rod inside the inner balloon. The corresponding photos of the actual coil are depicted in d, e, and f. A schematic cross section of the asymmetric ^1^H microstrip (g) shows its orientation and geometry. The copper colored (online version only) parts indicate the conducting line and ground plane. [Color figure can be viewed at http://www.wileyonlinelibrary.com]

**Figure 2:**
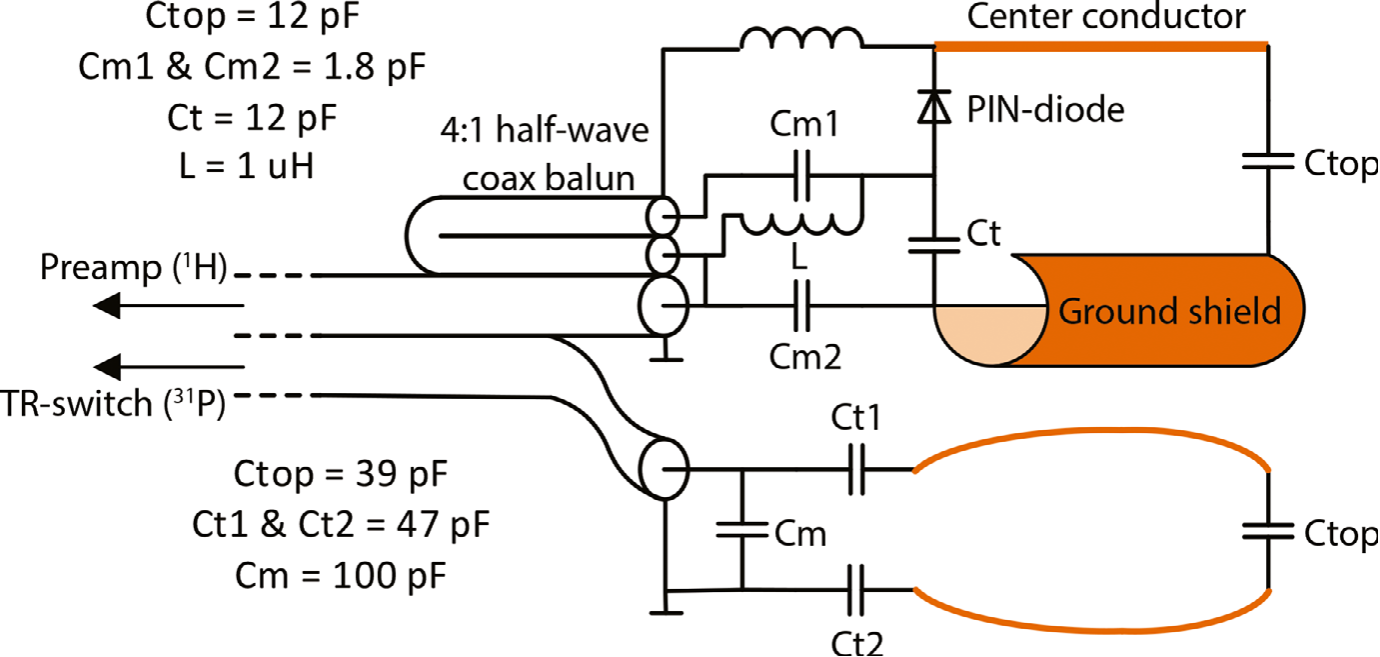
Schematic of the electrical circuit of the ^31^P/^1^H combined endorectal coil. Orange/brown colored (online version only) parts indicate the copper used for the actual ^1^H and ^31^P coils. [Color figure can be viewed at http://www.wileyonlinelibrary.com]

The mechanical housing and conductors of a 3T endorectal ^1^H receive (Rx) coil (MEDRAD, Pittsburgh, PA) were disassembled and equipped with a ^1^H receive element and a ^31^P transceiver loop coil. The ^1^H Rx element was placed directly on the plastic rod^16^ and consisted of an asymmetric microstrip of length 53 mm, with a distance of 4 mm between the conducting line and the ground plane [Fig. 1(a)]. By rotating the conducting line with respect to the axis of the plastic rod, the B_1_^‐^ profile was oriented in the same direction as the receive profile of the ^31^P loop coil. The coil was tuned and matched to 297.2 MHz and a series PIN diode was used for detuning the microstrip during transmit with the external ^1^H coil. The loop coil was tuned and matched to 50 Ω at 120.3 MHz for ^31^P at 7T and was mounted on top of the inner balloon (Fig. 1).^10^ In every patient measurement, after the final coil placement, the tuning and matching was checked using a network analyzer, as it slightly depends on the inflation of the inner balloon. The inner balloon was inflated using inert perfluoro‐polyether liquid (Fomblin, Solvay Solexis, Bollate, Italy). To eliminate any common modes a ^1^H cable trap was placed directly at the cable exit of the plastic rod. Finally, a balun was added to the ^1^H Rx element at the final part of the ^1^H cabling in the plastic rod (Fig. 2). The ^31^P transceiver loop was connected via a coaxial cable to a Tx/Rx switch with integrated small‐band low‐noise preamplifier (Ar^2^ Communication Products, Burlington US). The received signals from the ^1^H microstrip were routed through a separate coaxial cable to its own interface with integrated ^1^H preamplifier (Ar^2^ Communication Products, Burlington US). These electronics were used to interface the elements in the ERC to the MR system.

#### 2.2.2 ^1^H external array coil

The ^1^H external multitransmit RF body coil consisted of two arrays containing four microstrip elements with meanders.^10^, ^17^, ^18^ The arrays were put ventrally and dorsally of the body. The coil channels were powered by eight 1 kW amplifiers (LPPA 13080W, Dressler, Germany) and were connected using a separate Tx/Rx switch. The cable length between elements and switch box was adjusted to achieve preamplifier decoupling during reception.

#### 2.2.3 In vivo ^1^H performance of ^31^P/ ^1^H endorectal coil

To evaluate the *in vivo* performance of the asymmetric microstrip ^1^H receive element, we performed *in vivo* SNR measurements within the prostate to compare the SNR of the ERC with the SNR of the 8‐channel external body array (8CH). Gradient echo (GRE) imaging in transversal and sagittal orientation (TR = 25 ms, TE = 4.1 ms, field of view (FOV) = 261 × 380 mm^2^, matrix = 176 × 256, 3 slices, one average, bandwidth 241 Hz/pixel) of the healthy volunteer was performed with the ERC in situ, using the external body array for transmit and either only the 8‐channel external body array or only the ERC for receive. In the 8CH measurements, the images of the individual coil channels were added using the sum of squares method, after normalizing the individual images to their noise level which was determined within a region of interest (ROI) of air. For both measurements exactly the same measurement parameters were used. The SNR in pixel (x,y) of the ERC ($SNR_{x,y,ERC}$) and 8CH $(SNR_{x,y,8CH}$) measurements was determined by:

$$SNR_{x,y,ERC\left(8CH\right)}=\frac{Signal_{x,y}}{\sigma_{ERC\left(8CH\right)}}$$

Here, $\sigma_{ERC\left(8CH\right)}$ is an estimate for the noise within the prostate. A region of air was used for this estimation to reduce the influence of physiological noise and variation. $\sigma_{ERC\left(8CH\right)}$ was determined by correcting the standard deviation within the air ROI for the Rayleigh distribution in single channel ERC imaging and noncentral chi distribution in sum‐of‐squares multichannel 8CH imaging.^19^, ^20^

The ROI placement for noise calculation was the same for both measurements. The ratio of the SNR of the ERC and the external body array was determined by dividing the respective SNRs:

$$SNRratio_{x,y}=\frac{SNR_{x,y,ERC}}{SNR_{x,y,8CH}}$$

The SNR ratio image was filtered by convoluting the image with a 3 × 3 voxel wide block function.

### 2.3 Safety testing of the ^31^P/^1^H endorectal coil

Before performing any patient measurements, the setup was thoroughly validated for safety. The SAR safety levels of the setup were in part based on the simulations and measurements of a previously validated design by Kobus et al.^10^ They determined the safety levels of a ^31^P‐only ERC in combination with the same external body array as used in this study. Here we added a ^1^H Rx‐only microstrip to their ^31^P ERC design, which could influence the ^31^P loop coil performance. Because of the close proximity of the ERC to tissue, this could introduce safety concerns with respect to the maximum allowed ^31^P transmit power. To demonstrate the safety of the ^31^P/^1^H ERC, we performed electromagnetic field simulations of an experimental ^31^P/^1^H ERC design which we compared with a design similar to that of Kobus et al.^10^ In addition, we performed electromagnetic field measurements of this experimental ^31^P/^1^H ERC to validate the simulation results. The safety characteristics of the ^31^P/^1^H ERC were further investigated by performing SAR simulations in a more realistic prostate model and by performing *in vivo* temperature measurements.

Additional phantom measurements using a network analyzer to assess coupling between the ERC and the external coil array are presented in the Supporting Information S.1.

#### 2.3.1 Electromagnetic field simulations and phantom measurements

**Figure 3:**
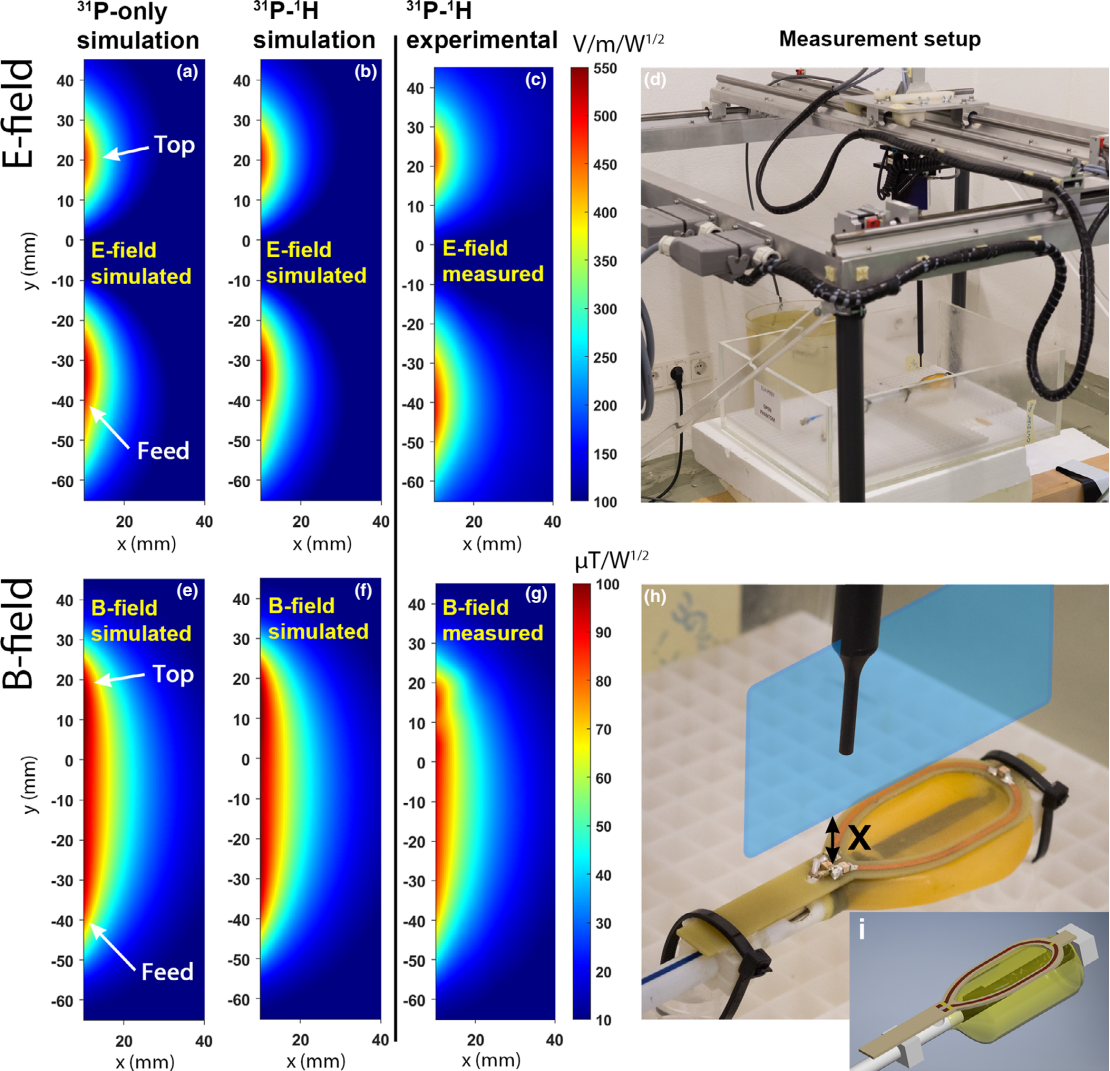
Electromagnetic field simulations of the ^31^P loop coil in transmit mode of the ^31^P‐only (E‐field: a, B‐field: e) and the ^31^P/^1^H (E‐field: b, B‐field: f) ERC. The corresponding experimental measurements of the ^31^P/^1^H ERC (E‐field: c, B‐field: g) are shown as well. An overview image (d) and a picture of the modified PCB coil (h) show the measurement setup and the sagittal plane (blue (online version only) plane) that was used to perform the measurements. The 3D model used for the simulations is shown in (i). X indicates the distance from the conductors in mm. Both the simulations and measurements were performed within the same plane. The feed of the coil was positioned at the bottom of the E‐ and B‐field maps, and the top of the coil was positioned at the top of the maps. For the actual measurements, the transparent box (d) containing the coil was filled with tissue simulating fluid and the loop was covered with a balloon. ERC, endorectal radiofrequency coil. [Color figure can be viewed at http://www.wileyonlinelibrary.com]

To promote reproducibility, the actual ^31^P loop coil was replaced by a stiff printed circuit board (PCB) plate with the same dimensions, in both simulations and measurements (Fig. 3). The electromagnetic fields of ^31^P/^1^H ERC were analyzed by performing Finite Integration Technique (FIT) simulations (CST Studio Suite 2011, Darmstadt, Germany) at 120.3 MHz of a three‐dimensional (3D) model (Autodesk Inventor, San Rafael, USA) of the ^31^P/^1^H endorectal coil and a similar ERC without the ^1^H channel (^31^P‐only ERC) resembling the design by Kobus et al.^10^ In the simulations of the 3D models of the coils, the coils were surrounded by a phantom fluid with a relative permittivity of ϵ_r_ = 79 and a conductivity of σ = 0.47 S/m at a frequency of 120.3 MHz. Additional measurements were performed of the ^31^P/^1^H ERC using a setup with field probes (Schmid & Partner Engineering AG (SPEAG), Zürich, Switzerland, probes H3DV7 & ES3DV2) to collect E‐ and B‐field measurements in the sagittal plane in a phantom filled with liquid (ϵ_r_ = 79, σ = 0.49 S/m, measured with an Agilent 85070E dielectric probe kit at 120 MHz). To stay within the calibration range of the probes, two different power settings were used: 0.01 W for the B‐field and 0.05 W for the E‐field measurements. The measurements were performed at 120 MHz in a 2D plane using a sampling density of one sample per 5 × 5 mm^2^ area. A sagittal plane of 150 × 30 mm^2^ was sampled in the midline of the coil, 10–40 mm above the conductors.

#### 2.3.2 SAR simulations

**Figure 4:**
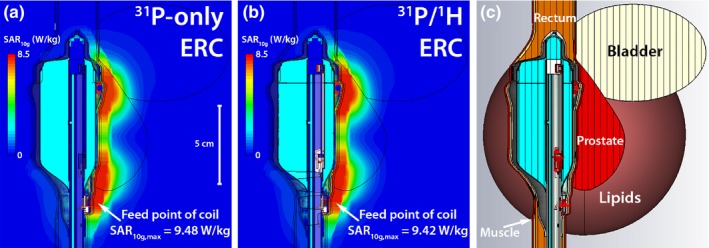
SAR simulations of the ^31^P transceiver loop coil in transmit mode of the ^31^P‐only ERC **(a)** and the ^31^P/^1^H ERC **(b)**. The ERC model with the actual ^31^P loop coil (Fig. 1) was used for the simulations. The simulations were performed with a model **(c)** to get a realistic simulation environment (prostate: ϵ_r_ = 72.8, σ = 0.923 S/m, ρ = 1045 kg/m^3^; bladder: ϵ_r_ = 22, σ = 0.297 S/m, ρ = 1035 kg/m^3^; lipids: ϵ_r_ = 12.4, σ = 0.0694 S/m, ρ = 911 kg/m^3^; rectum: ϵ_r_ = 64, σ = 0.717 S/m, ρ = 1045 kg/m^3^, muscle: ϵ_r_ = 64, σ = 0.717 S/m, ρ = 1090 kg/m^3^). ERC, endorectal radiofrequency coil. [Color figure can be viewed at http://www.wileyonlinelibrary.com]

Electromagnetic field simulations (FIT) of the ^31^P loop coil at 120.3 MHz in a more realistic prostate model were performed to determine the SAR levels at 1 W transmit power. The ^31^P/^1^H ERC was compared to the ^31^P‐only ERC in a model incorporating the different surrounding tissues (Fig. 4). Both were simulated with the actual ^31^P loop coil model (Fig. 1). The permittivity and electric conductivity values were obtained from literature.^21^

#### 2.3.3 In vivo temperature measurements

**Figure 5:**
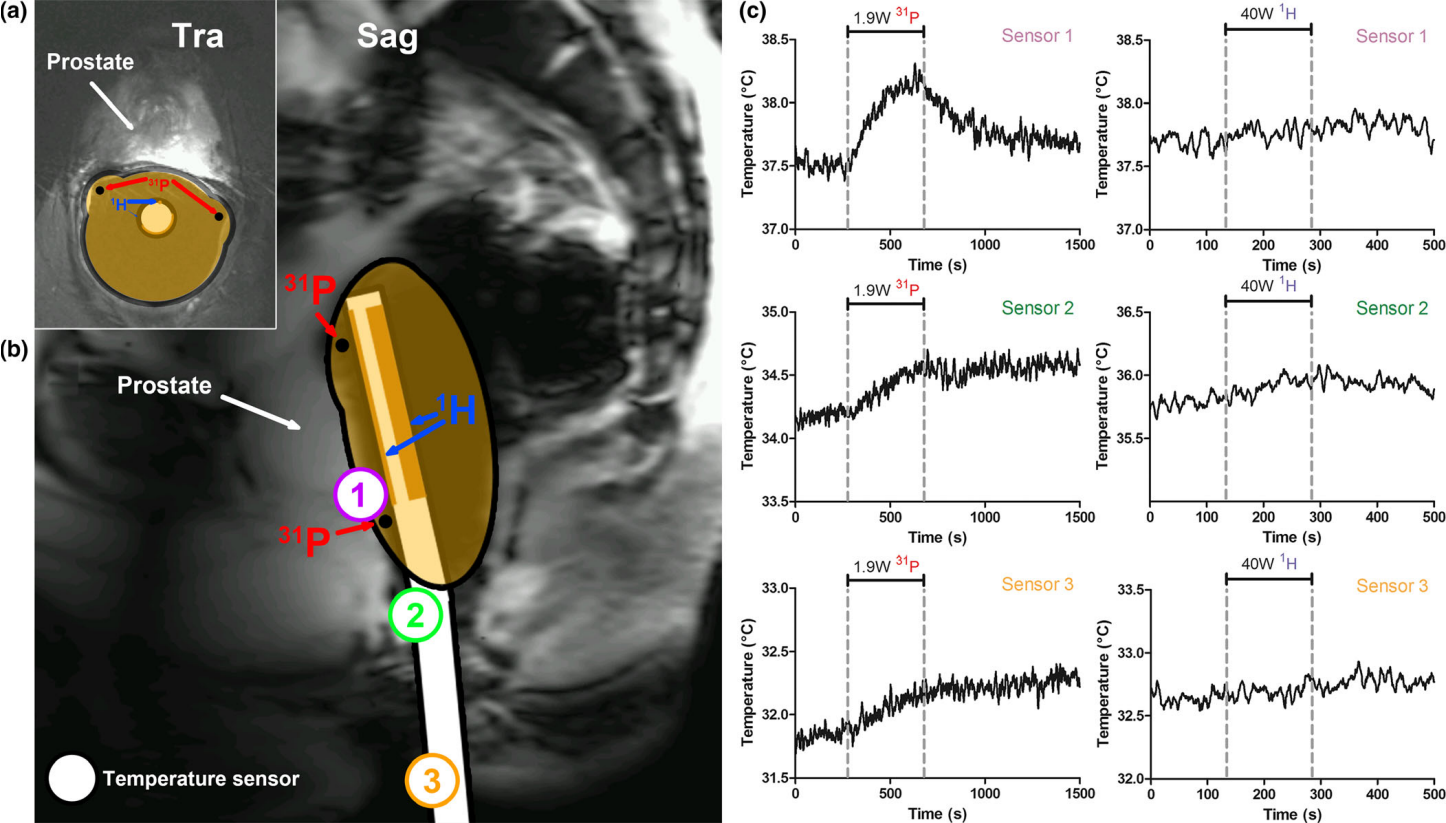
*In vivo* transversal (a) and sagittal (b) image of the prostate of a volunteer, illustrating the standard endorectal coil position during prostate MR examinations. The position of the temperature sensors that were placed during the *in vivo* temperature measurements are depicted on the sagittal image (b). The temperature during the ^31^P (at 1.9 W, left graph) and ^1^H (at 40 W, right graph) transmit period is depicted for sensor 1, 2, and 3 (c). The dashed lines indicate the period in which power was transmitted (6 min for ^31^P and 2.5 min for ^1^H). MR, magnetic resonance. [Color figure can be viewed at http://www.wileyonlinelibrary.com]

*In vivo* temperature measurements were performed with the complete setup: the ^31^P/^1^H ERC in combination with the external ^1^H body array. Three thermocouples were attached to the ERC at different locations (Fig. 5). One temperature sensor was placed at the feed point of the coil, the location of the hotspot in previous SAR simulation by Kobus et al,^10^ and the other two sensors were attached to the cable to ensure no safety compromising common modes were present. The thermocouple at the feeding point was attached to the outer side of the external balloon and a thin condom was used to cover the ERC and thermocouples. The insulating effect of this thin latex layer was expected to be of minimal influence and was therefore neglected. Two different safety tests were performed. The ERC was applied as usual and for the first test a time‐averaged input power of 1.9 W^10^ was used for a duration of 6 min on the ^31^P channel. For the second test the external ^1^H body array was used with a power of 40 W for 2:30 min to test for any coupling between the body array and the ERC.

### 2.4 Patient measurements

To demonstrate the feasibility of the complete setup, all patient measurements were performed with the external body array coil for ^1^H transmit only and the ^31^P/^1^H ERC for ^1^H receive and ^31^P transmit and receive (see Figure S‐2 in Supporting Information S.3 in for a schematic overview of the *in vivo* setup). After a localizer and B_0_ shimming, B_1_^+^ shimming was performed by using the B1TIAMO technique 22 to acquire absolute B_1_^+^ maps and by maximizing the phase coherence of the individual coil channels in an ROI within the prostate. ^1^H T_2_‐weighted imaging and diffusion‐weighted imaging were then performed with an in‐plane spatial resolution of respectively 0.43 × 0.43 mm^2^ and 1.75 × 1.75 mm^2^.

Proton MRSI was performed using a PRESS‐like sequence with RF refocusing pulses that are both spectrally and spatially selective, such that the signal of a volume of interest (VOI) is received only from the spectral region of interest (2.3–3.3 ppm).^23^ ^31^P MRSI was performed using a 3D phase‐encoded pulse‐acquire sequence with a nonselective BIR‐4 excitation pulse^9^ (see Table 1 for further imaging and spectroscopy details). The nuclear Overhauser effect was used with continuous low‐power irradiation of the water resonance to enhance ^31^P SNR.^15^ ^31^P MRSI data was fitted using Metabolite Report (Siemens Healthineers Erlangen, Germany). PE, PC, GPE, and GPC were modeled as a triplet of three Gaussian peaks with a separation equal to the J‐coupling (6–7.1 Hz) and amplitudes in a 1:2:1 ratio. The PCr lineshape, which was fitted with a single Gaussian shape, was used as a constraint to the linewidth for all signals.^9^ The spectra shown in this work were corrected for constant and linear phase, an exponential time filter (40 ms for ^31^P and 150 ms for ^1^H) was applied and the spectra were zero‐filled to 2048 points. In the ^31^P spectra the PCr signal was referenced to 0.0 ppm and in the ^1^H spectra water was referenced to 4.7 ppm.

**Table 1:** Sequence parameters

	T_2_W	DWI	^31^P MRSI^b^	^1^H MRSI
Sequence type	Turbo‐Spin‐Echo	EPI (RESOLVE); b0, b100, b400, and b800	3D MRSI with non‐selective BIR‐4 excitation	PRESS‐like with spectrally and spatially selective RF refocusing pulses
FOV	111 × 111 mm^2^	154 × 154 mm^2^	a): 120 × 100 × 100 mm^3^ b): 120 × 120 × 120 mm^3^	84 × 70 × 70 mm^3^
Matrix	256 × 256	88 × 88	a): 12 × 10 × 10 b): 10 × 10 × 10	12 × 10 × 10
Voxel size^a^	0.43 × 0.43 × 3 mm^3^	1.75 × 1.75 × 3 mm^3^	a): 4.9 cc b): 9.1 cc	1.4 cc
Slices	19	19	n.a.	n.a.
Slice thickness	3	3	n.a.	n.a.
TR (ms)	4250	4400	1500	1000
TE (ms)	84	68	n.a.	135
Bandwidth (Hz)	106 (per pixel)	1015	10000 (spectral)	2400 (spectral)
Vector size	n.a.	n.a.	2048	1024
Averages	1	1	4 (weighted)	1
Acquisition time (min:sec)	1:59	4:34	13:09	7:01
Flip angle (⁰)	n.a.	n.a.	45	n.a.
Filter	Prescan normalization filter	n.a.	100% Hamming filter	50% Hamming filter

DWI, diffusion‐weighted imaging; MRSI, magnetic resonance spectroscopic imaging.

a For the MRSI measurements, this was based on the true voxel size based on voxel volume within the 50% range of the maximum of the point spread function after correcting for spatial filter and sampling scheme.

b Two different measurement parameter sets were used: set a) and set b).

## 3 Results

The ^31^P/^1^H ERC was constructed according to the circuit and model specifications (Figs. 1 and 2) and all validations and *in vivo* experiments were performed successfully.

Simulations of the B_1_^−^ profile of the ^1^H microstrip were performed and are shown in the Figure S‐1 in Supporting Information S.2.

### 3.1 Safety

The electromagnetic field simulations to test the safety when transmitting at ^31^P showed no differences between the ^31^P‐only ERC by Kobus et al.^10^ and the ^31^P/^1^H ERC proposed in this work (Fig. 3). The hotspot with respect to the electric field in both the ^31^P‐only and the ^31^P/^1^H ERC was identified at the same location at the feed point of the coil. Both the E‐ and B‐field simulations corresponded well with the actual measurements of the ^31^P/^1^H ERC, yielding similar E‐ and B‐field peak locations and intensities. The SAR simulations in the prostate tissue model also showed no substantial differences between the two coils. Maximum SAR_10g_ values of 9.48 W/kg for the ^31^P‐only ERC and 9.42 W/kg for the ^31^P/^1^H ERC were found at the feed point of the coil when transmitting with the ^31^P loop coil at 1 W (Fig. 4).

When transmitting on ^31^P at 1.9 W power, the *in vivo* temperature measurements showed a maximum temperature increase of approximately 0.7°C over 6 min at the feed point of the coil, converging to a plateau in the second half of the 6‐min period. The maximum temperature increase measured at the sensors located on the cable was < 0.5°C. When transmitting with the external ^1^H body array at 40 W for 2:30 min, the temperature increase was less than 0.3°C in any of the temperature sensors, indicating that there is no significant coupling between the external array and the ERC (Fig. 5).

There was no evidence for any significant coupling between the ^1^H ERC channel, the ^31^P ERC channel and the external body array in the phantom measurements with the network analyzer. There was also no influence when manipulating the cables (Table S‐1 in Supporting Information S.1).

### 3.2 Volunteer and patient measurements

**Figure 6:**
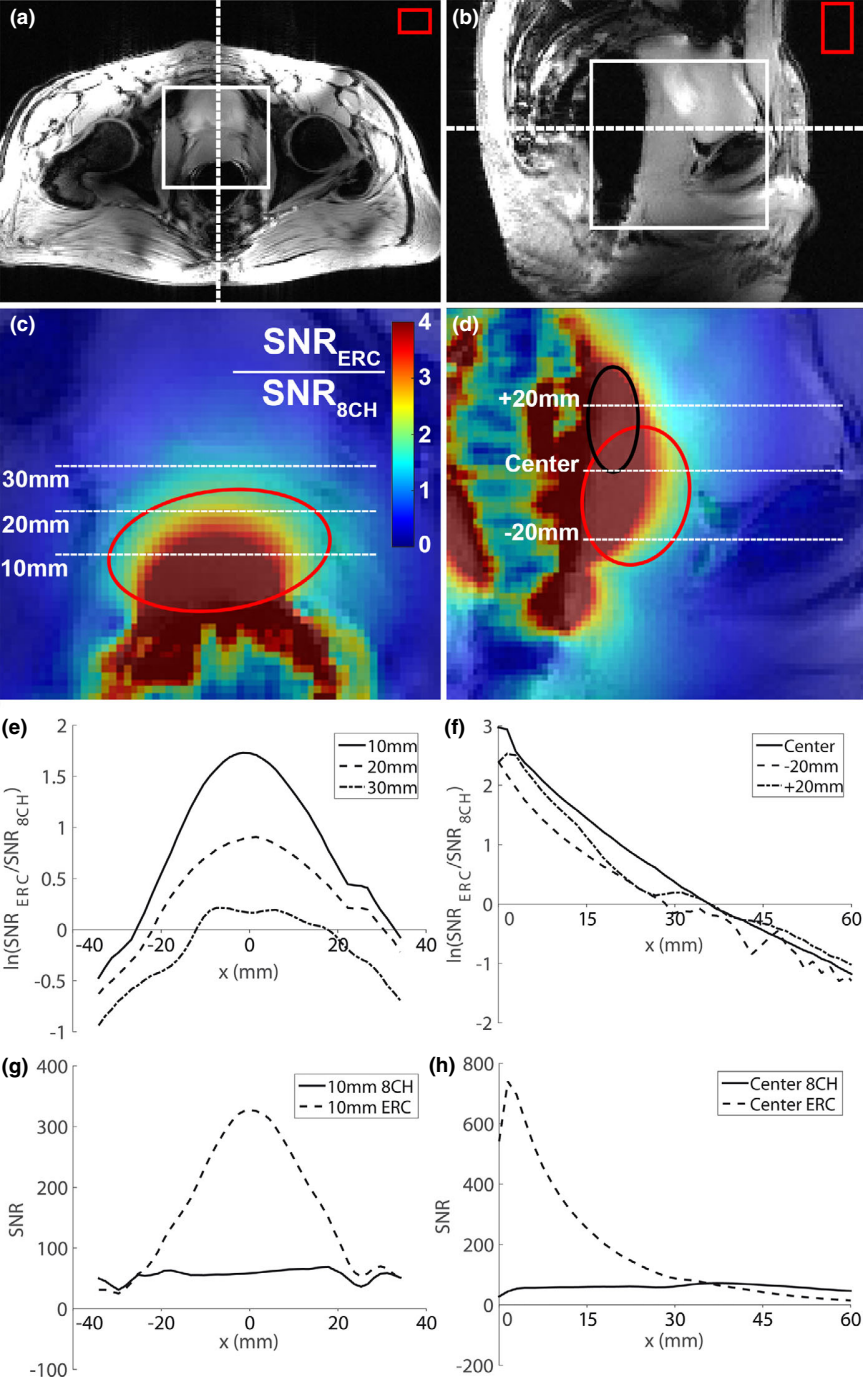
Gradient echo imaging of the prostate of a healthy volunteer in transversal (a) and sagittal (b) orientation using the 8‐channel external body array (8CH). The red circles (online version only) indicate the outline of the prostate and the black circle (online version only) indicates the outline of the seminal vesicles. The dashed lines indicate the location of the transversal and sagittal slice with respect to each other (a, b), the white box indicates the position of the respective colormap image (c, d) and the red box indicates the ROI in air that was used to estimate the noise. A map of the ratio of the SNR (SNR_ERC_/SNR_8CH_) obtained with only the ERC’s ^1^H asymmetric microstrip and only the 8CH array was determined and overlaid on the GRE transversal (c) and sagittal (d) imaging. The cross section of the SNR ratio was obtained at three different locations in the left right direction on the transversal imaging at 10, 20, and 30 mm anterior to the endorectal coil (e). The cross section of the SNR ratio was also determined at three different locations on the sagittal images, through the center of the ERC and 20 mm superior and inferior to center of the ERC (f). The natural logarithm of the SNR ratio (ln(SNR_ERC_/SNR_8CH_)) was plotted with respect to the location. The first pixel anterior to the ERC with actual signal was taken as x = 0 for the sagittal cross section and the center of the prostate was used as x = 0 in the transversal cross section. The absolute SNR of the ERC and the 8CH are also shown for the cross section at 10 mm anterior to the ERC on the transversal image (g) and for the central cross section on the sagittal image (h). ERC, endorectal radiofrequency coil; ROI, region of interest; SNR, signal‐to‐noise. [Color figure can be viewed at http://www.wileyonlinelibrary.com]

**Figure 7:**
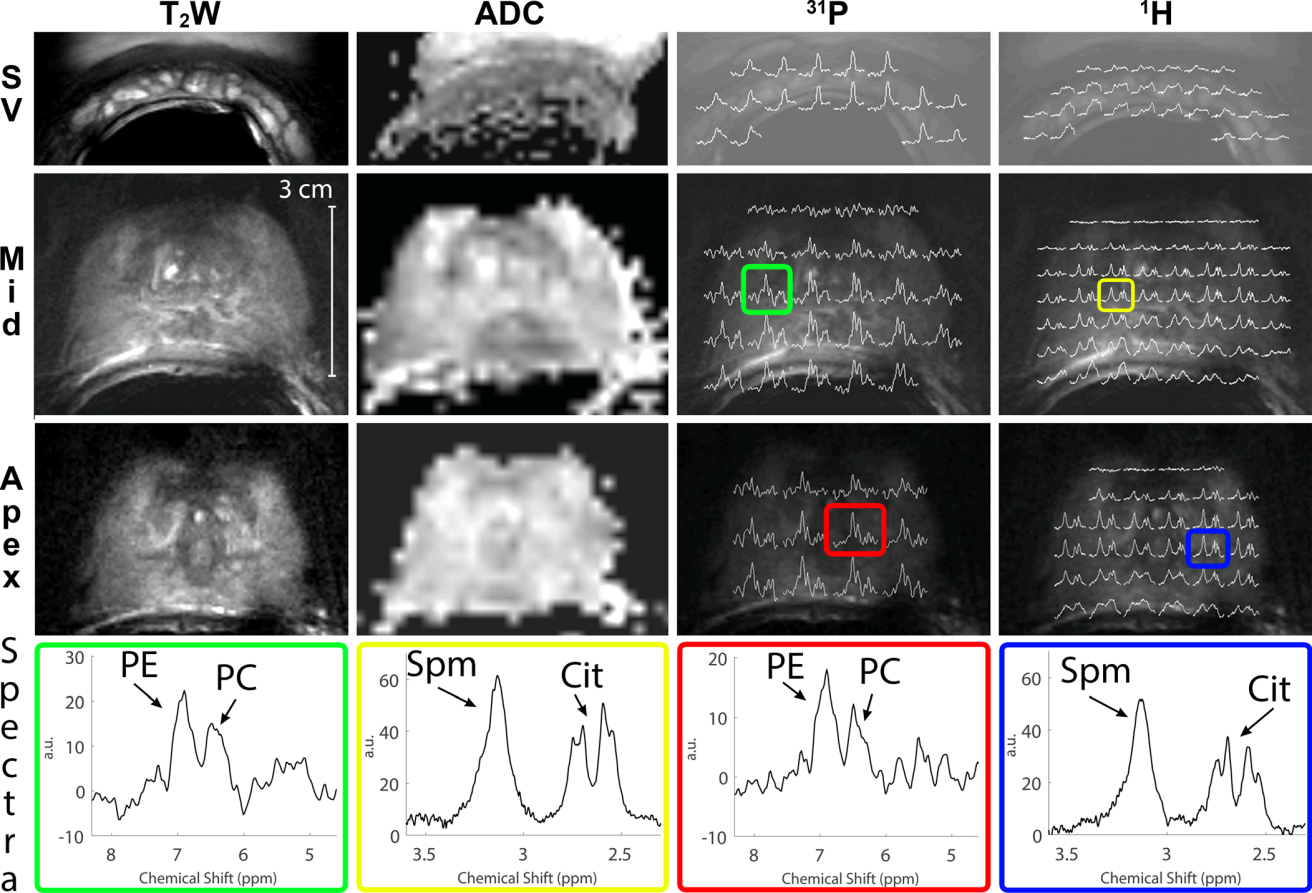
T_2_W imaging, DWI ADC maps, ^31^P MRSI spectral maps (^31^P), and ^1^H MRSI spectral maps (^1^H) of three different regions of a healthy volunteer (40 yr, 83 kg): the apex, mid‐prostate, and the seminal vesicles (SV). The spectral maps of ^31^P MRSI were taken of the spectral region 4.6–8.3 ppm, showing the PE signal (left peak) and PC signal (right peak). In some spectra, the inorganic phosphate (Pi) signal is also visible on the right of the spectrum. The ^1^H spectra are shown of the spectral region 2.3–3.6 ppm, showing mainly spermine (left side of the spectrum) and citrate (right side of the spectrum). ^31^P MRSI measurement set a) was used. To provide a more detailed view, four of these spectra are shown separately (Spectra), two for ^31^P (green and red), and two for ^1^H (yellow and blue). The location of these spectra is indicated with colored boxes (online version only) on the spectral maps. DWI, diffusion‐weighted imaging; MRSI, magnetic resonance spectroscopic imaging. [Color figure can be viewed at http://www.wileyonlinelibrary.com]

Using the external body array for ^1^H transmit resulted in a homogeneous flip angle distribution over an ROI in the prostate of the volunteer of 78.5 ± 3.9° (Figure S‐2 in Supporting Information S.3). In combination with the high local sensitivity of the ERC’s ^1^H Rx channel, this allowed high resolution T_2_W and DW imaging without artifacts (Fig. 7, Figure S‐3 in Supporting Information S.4). The ERC’s ^1^H asymmetric microstrip showed improved SNR in the prostate of the healthy volunteer when compared with the external array, up to 3.5 cm anterior to the ERC at the center of the microstrip and 3 cm when 20 mm superior or inferior to the center of the microstrip [(Fig. 6(d) and 6(f)]. In the midline through the ERC, 1 cm into the prostate, the SNR (mean ± standard error of the mean) over the center ten voxels was 316 ± 7 for the ERC microstrip and 59 ± 0.6 for the 8CH array. Due to the asymmetric microstrip design, the receive profile is symmetric with respect to and oriented toward the prostate [Fig. 6(c)], (Figure S‐1 in Supporting Information S.2). Thirty millimeters anterior to the ERC, the ERC had a higher SNR than the 8CH within a range of ≥ 30 mm in the left‐right direction [Fig. 6c and 6(e)].

**Figure 8:**
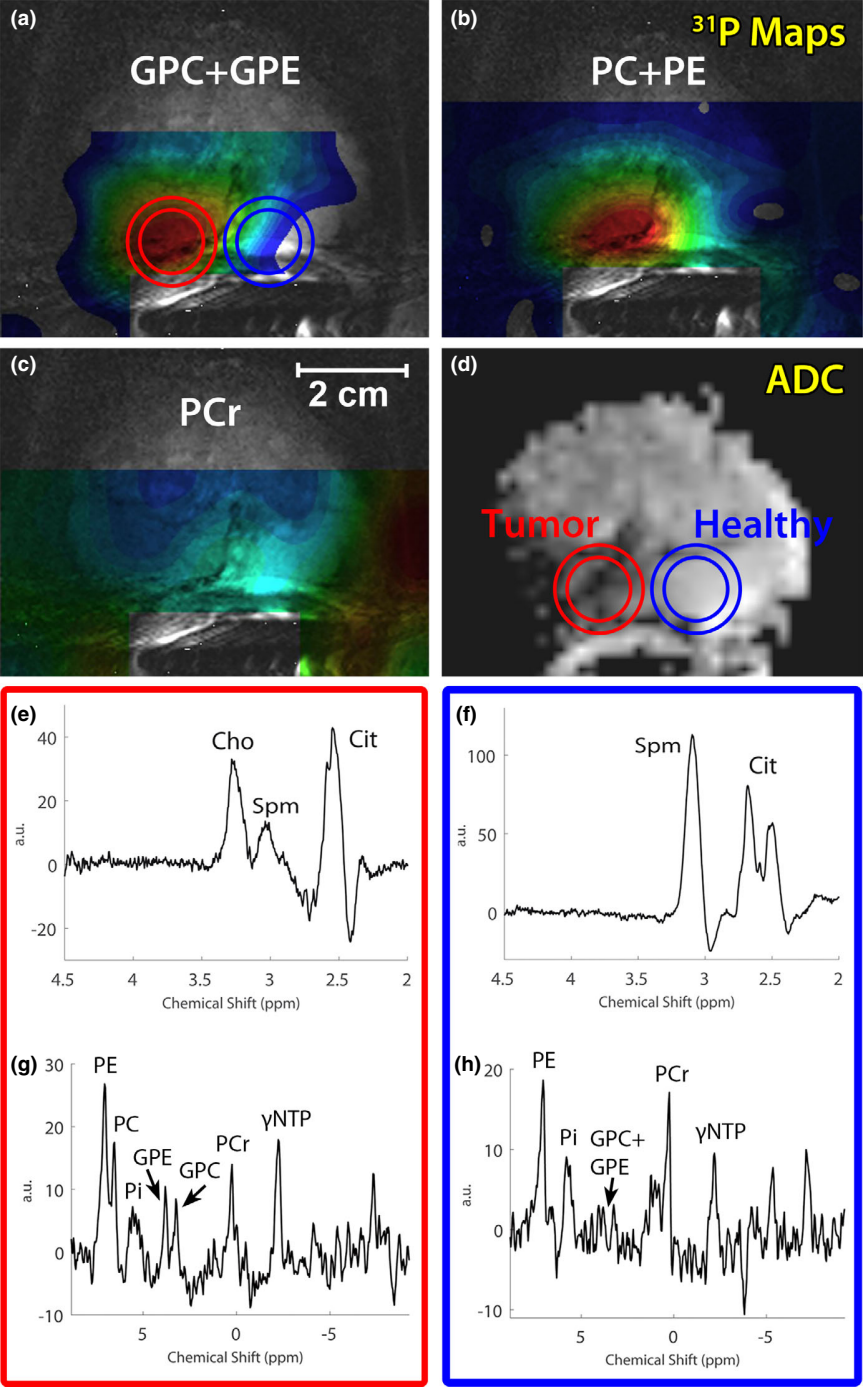
GPC + GPE, PC + PE, and PCr metabolite maps of a patient with metastatic Gleason 4 + 4 prostate cancer (67 yr old, 88 kg, MR‐TRUS guided biopsy) (a,b, and c) overlaid over a T_2_‐weighted image. The corresponding ADC map (d) is also shown, indicating a large prostate cancer lesion on the right side of the prostate. ^1^H (e,f) and ^31^P (g,h) spectra are shown of the cancer lesion (red circle) as well as a location within healthy tissue (blue circle) (online version only). ^31^P MRSI measurement set a) was used. GPC, glycerophosphocholine; GPE, glycerophosphoethanolamine; MRSI, magnetic resonance spectroscopic imaging; PC, phosphocholine; PE, phosphoethanolamine.

We were able to detect prostate cancer lesions in the three patients with the imaging sequences, such that voxels with ^1^H and ^31^P spectra could be localized within these lesions. Prostate cancer lesions were verified by MR‐guided biopsy (one patient), MR‐TRUS guided biopsy (one patient) or TRUS biopsy and 3T clinical MRI results (one patient). The ERC’s sensitivity profile was wide enough to perform T_2_W and DW imaging of the seminal vesicles, apex of the prostate, and anterior regions of the prostate (Fig. 7), even in a relatively large prostate (Figure S‐3 in Supporting Information S.4). Due to the overlap between the ^31^P and ^1^H sensitivity profiles, ^31^P spectroscopy of those regions can be obtained as well with sufficient SNR to measure PE and PC in a healthy volunteer or patient (Fig. 7 *,* Fig. 8). The ^1^H spectral map shows adequate SNR for citrate and spermine from apex to base in the prostate. The line widths achieved after B_0_ shimming within a VOI around the prostate ranged from 30–60 Hz. Close to the ERC and in the seminal vesicles the spectral map shows wider peaks because those areas are generally harder to B_0_ shim properly. This was less noticeable in the ^31^P spectra due to the larger ppm difference of the ^31^P signals. For both ^31^P and ^1^H spectroscopy the signal drops anteriorly in prostate.

^31^P spectroscopic imaging was obtained successfully in the volunteer and in all patients. PE, PC, PCr, and γ‐ and αNTP were detectable in spectra that were located within the prostate. Similar to previous results^9^ the PDEs were occasionally within detectable ranges, and if so, they were well separable (Fig. 8). ^1^H spectroscopy showed well distinguishable tCho and Spm signals and also showed Cit which occasionally suffered from lipid contamination. High spermine signals were present in ^1^H spectra (Fig. 8, Figure S‐3 in Supporting Information S.4).

In one of the patients with a high‐grade Gleason 4 + 4 prostate cancer lesion the GPC, GPE, and PC levels were clearly identifiable and elevated in the spectra located within the cancer lesion, whereas these signals were almost absent in contralateral healthy prostate tissue (Fig. 8). PCr, which is mostly present in muscle tissue, could serve as an indicator for the ^31^P coil sensitivity profile due to its assumed symmetric distribution over the prostate and nearby smooth muscles. As PCr was highest on the healthy side of the prostate, the GPE, GPC, and PC elevations in the lesion were not caused by any asymmetry in the coil profile, but were due to real locally increased metabolite levels. The increase in ^31^P metabolites in tumor tissue corresponded with an elevated ^1^H tCho peak, which was absent on the contralateral side of the prostate. Other patients did not show detectable GPC or GPE within the cancer lesion (Figure S‐4 in Supporting Information S.4).

Please refer to the Supporting Information S.4 for additional patient measurements.

## 4 Discussion

^31^P spectroscopic imaging of the prostate is only possible with an ERC at an ultra‐high field strength. When adding a ^1^H receive channel to the ERC and combining it with an external body array coil for ^1^H transmit, ^31^P spectroscopic imaging can be performed within the same MR examination as high‐resolution prostate imaging and ^1^H spectroscopic imaging.

### 4.1 Safety

To validate the safety of our setup, we used the results of previous work by Kobus et al^10^ with a ^31^P Tx/Rx ERC combined with an external body coil. Their work showed no substantial coupling between the ERC and the external body array. Furthermore, when transmitting at ^31^P with a power of 1.9 W their simulations stayed within the SAR_10g_ limit of the first level operation mode in the hotspot at the feed of the coil.

In our proposed ^31^P Tx/Rx – ^1^H Rx endorectal coil, we added a ^1^H receive microstrip to the ^31^P Tx/Rx ERC by Kobus et al.^10^ This addition may pose safety concerns for the ^31^P transmit safety levels. We performed electromagnetic field and SAR simulations of both the ^31^P‐only ERC by Kobus et al and our proposed design with an additional ^1^H microstrip and found no influence of the ^1^H microstrip on the simulation results or local hotspot locations. We also validated these results with experimental phantom measurements. These results indicate that we can use the SAR safety levels of Kobus et al^10^ for our design and that our design stays within the local SAR_10g_ limit of first level operation mode when transmitting at 1.9 W on ^31^P. Moreover, new *in vivo* temperature measurements of the proposed ^31^P/^1^H ERC also showed a temperature increase < 1°C in the local hotspot when transmitting at this power level, again indicating safe power levels.

### 4.2 *In vivo* measurements

Kobus et al^10^ and Lagemaat et al^9^ used a measurement setup with an external body array in combination with a ^31^P‐only ERC, which allowed them to obtain imaging and ^31^P spectroscopy. No ^1^H spectroscopic imaging was performed, as this is challenging without a local receive coil due to SNR restrictions. In this work we equipped the ERC with a ^1^H Rx element to substantially increase local receive sensitivity. Not only did this facilitate the acquisition of ^1^H MRSI, it also enabled high resolution T_2_W imaging and DWI with a zoomed field‐of‐view of the prostate. The imaging resolution was substantially higher than the reported spatial resolution of 0.75 × 0.75 × 3 mm^3^ in earlier work at 7T without an ERC^24^ and similar to the resolution of 0.4 × 0.4 × 3 mm^3^ in earlier work at 3T with a dedicated ^1^H ERC.^25^ This resolution is possible due to the SNR increase that the ERC ^1^H microstrip offered in the prostate. Up to 3.5 cm anterior to the ERC, the ERC has a higher SNR than the 8‐channel external body array, which is comparable to previously published work on ^1^H imaging at 7T.^26^ When combined with the external body array for receive, the ERC would provide additional performance up to about 5 cm in the prostate (when taking the − 0.5 logarithmic SNR ratio criterium for added benefit in Fig. 6). The SNR measurements were performed in one healthy volunteer of average body composition and we corrected for differences in noise distribution between the reconstruction algorithms used, to perform a fair comparison. The SNR gain of the ERC with respect to a multichannel array surely also depends upon the exact body composition: in larger body circumferences the elements of the external coil array are further away from the prostate, decreasing their SNR within the prostate, increasing the relative SNR contribution from the ERC over a larger area. The opposite holds for smaller body compositions. The overlap in the ^31^P and ^1^H sensitivity profile of the ERC allowed combining ^1^H with ^31^P MRSI in the same patient, enabling us to correlate tCho signals to individual phosphorus containing choline signals. Because of the SNR constraints of spectroscopy, the ERC might not be optimal for assessing the anterior regions of (very) large prostates, as the sensitivity profile of an ERC drops significantly at large distances.

Because of the increased SNR due to the use of 7T MRI in combination with the nuclear Overhauser effect, ^31^P spectroscopic imaging was obtained with high quality spectra in all patients, using a spectroscopic imaging method to accurately localize signals. Although the true voxel size was 4.9 or 9.1 cc, it allows voxel localization with respect to the T_2_W and DWI images within or covering suspicious lesions, instead of relying on coil profile‐based localization with an unlocalized pulse sequence. Using a ^31^P/^1^H ERC without external multitransmit array coil also enables the acquisition of ^31^P and ^1^H MRSI. However, one would need to revert to the use of many adiabatic RF refocusing pulses and T_2_‐weighted MRI and DWI becomes particularly problematic with the use of only a small endorectal coil 12.

^1^H MRSI was also obtained with a high SNR and with well distinguishable tCho and Spm signals, but it suffered from lipid contamination in some cases. This is most likely caused by B_0_ inhomogeneities, the small chemical shift separation between the citrate and lipid signals and the spectral selectivity of the spectral‐spatial pulses of the ^1^H spectroscopy sequence. ^1^H MRSI was performed with a corrected voxel size of 1.4 cc, enabling voxel localization within or covering lesions. The spectral spatial refocusing pulses selectively inverted the 3.1 ppm resonance of Spm, refocusing its J‐coupling at the echo time,^23^ resulting in high spermine signals that might yield additional biological information.^1^, ^27^

In the seminal vesicles, an increased PC/PE ratio and a high tCho signal with an almost absent Spm peak were found (Figure S‐3 in Supporting Information S.4). In one particular case (Fig. 8) with an aggressive Gleason 4 + 4 prostate cancer with bone metastases, increased levels of total choline were observed in the cancer lesion. The elevated tCho correlated with increases in GPE, GPC, and PC levels and suggests an increased degradation of membrane phospholipids.^7^

Although there is a correlation between ^31^P and ^1^H choline signals, it appears as though the increase in ^31^P metabolites cannot completely account for the large difference in tCho levels observed between cancer and normal tissue. This indicates that also other overlapping signals, such as free choline, change significantly, but drawing conclusions about specific free choline levels may be difficult using the current setup. Due to the ERC profile, absolute quantification of the metabolites is difficult and necessitates the use of metabolite ratios or coil profile corrections to draw more quantitative conclusions. For ^31^P spectroscopy, relevant ratios would be PDE/PME, PC/PE, or PE/(PDE + PME).^9^ For ^1^H spectroscopy tCho/Spm could be used, as tCho tends to be elevated in cancer lesions, whereas spermine tends to decrease in cancer tissue.^27^

## 5 Conclusion

Using a ^31^P Tx/Rx ^1^H Rx endorectal coil in combination with an external multitransmit ^1^H body array enables high resolution multiparametric MR imaging and ^1^H and ^31^P spectroscopy of the prostate at 7T. The setup does not pose any safety issues and provides superior ^1^H SNR locally in the prostate in comparison with a ^1^H external body array alone. The clinical data shown in this study are anecdotal but they illustrate how the proposed setup would be particularly suitable to combine anatomically and functionally with metabolic imaging in prostate cancer.

## Conflicts of interest

The authors have no relevant conflicts of interest to disclose.

## Supporting information


**Figure S‐1: **B_1_^−^ simulations of the ^1^H Rx asymmetric microstrip (a,c,e) and a loop coil with similar length (b,d,f).
**Figure S‐2: **A schematic transversal overview of the complete *in vivo *measurement setup, including a transversal flip angle map through the abdomen (volunteer, age 40 years, weight 83 kg) when transmitting with the combined 8‐channel coil array after B_1_^+^ shimming.
**Figure S‐3: **Transversal T_2_W imaging of a patient (60 years old, 97 kg) in the seminal vesicles (a), the mid‐prostate (b) and the apex (c) of the prostate.
**Figure S‐4: **ADC map (a), T_2_W image (b) and ^31^P MRSI spectrum (c) of a patient (63 years, 83 kg) with prostate cancer (Gleason 3+4).
**Table S‐1: **Coupling between ^31^P/^1^H endorectal coil and external ^1^H body array in dB.
