# Supplementary material for: A multitransmit external body array combined with a 1H and 31P endorectal coil to enable a multiparametric and multimetabolic MRI examination of the prostate at 7T
Source: Med Phys. 2019 Aug 1;46(9):3893–905. doi: 10.1002/mp.13696 (PMC6852321; doi:10.1002/mp.13696)
Supplement: Supplementary file 1 — Figure S‐1: B1 − simulations of the 1H Rx asymmetric microstrip (a,c,e) and a loop coil with similar length (b,d,f). Figure S‐2: A schematic transversal overview of the complete in vivo measurement setup, including a transversal flip angle map through the abdomen (volunteer, age 40 years, weight 83 kg) when transmitting with the combined 8‐channel coil array after B1 + shimming. Figure S‐3: Transversal T2W imaging of a patient (60 years old, 97 kg) in the seminal vesicles (a), the mid‐prostate (b) and the apex (c) of the prostate. Figure S‐4: ADC map (a), T2W image (b) and 31P MRSI spectrum (c) of a patient (63 years, 83 kg) with prostate cancer (Gleason 3+4). Table S‐1: Coupling between 31P/1H endorectal coil and external 1H body array in dB. [file MP-46-3893-s001.pdf]

**Supplemental material**

### *S.1 $^{31}$P Tx/Rx -$^{1}$H Rx endorectal coil and 8-channel $^{1}$H array coil coupling*

To evaluate whether there was any coupling between the endorectal coil and the external body array, we determined the respective scattering parameters (S-parameters). We determined the $S_{11}$ of the external body array for each channel with and without the $^{31}P/^{1}H$ endorectal coil. In addition, the $S_{21}$ was determined between the external body array and the $^{31}P$ and the detuned $^{1}H$ receive element of the $^{31}P/^{1}H$ endorectal coil. For these measurements the external body array was positioned around a body phantom (oval shaped; 28x19.5 cm and height 70 cm) filled a fluid ($\epsilon_r = 43$ and a conductivity of $\sigma$ = 0.72 S/m at a frequency of 297 MHz). When the endorectal coil was included in the measurements it was placed in the center of the body phantom. All open coil connections were terminated with 50 Ω.

The $S_{21}$ coupling coefficient between the external body array channels and the endorectal coil was < -53 dB for the $^{31}P$ element and < -67 dB for the detuned $^{1}H$ element. No substantial influence of the endorectal coil was found on the reflection coefficients $S_{11}$ of the external body array. There was also no influence when manipulating the cables. (Supplemental Table S-1).

**Supplemental Table S-1**: Coupling between $^{31}P/^{1}H$ endorectal coil and external $^{1}H$ body array in dB. $S_{11}$ of $^{1}H$ external body array was determined with and without endorectal coil placed in the center of the setup. The $S_{21}$ was determined between the external body array and the $^{31}P$ or $^{1}H$ channel of the $^{31}P/^{1}H$ endorectal coil.

| External coil channel | $S_{12}$ $^{31}$P endo | $S_{12}$ $^{1}$H endo | $S_{11}$ with endo | $S_{11}$ without endo |
|---|---|---|---|---|
| 1 | -60 | -80 | -22 | -22 |
| 2 | -60 | -70 | -23 | -23 |
| 3 | -53 | -67 | -31 | -36 |
| 4 | -55 | -70 | -20 | -20 |
| 5 | -64 | -80 | -9 | -9 |
| 6 | -59 | -70 | -18 | -18 |
| 7 | -60 | -70 | -17 | -17 |
| 8 | -60 | -80 | -10 | -10 |

### *S.2 Simulations of the $^{1}H$ receive asymmetric microstrip.*

To assess the performance of the asymmetric microstrip $^{1}H$ receive element of the $^{31}P/^{1}H$ endorectal coil, Finite Integration Technique (FIT) simulations (CST Studio Suite 2011, Darmstadt, Germany) of a 3D model (Autodesk Inventor, San Rafael, USA) of the endorectal coil at 297.2 MHz were performed. To ascertain that our practical approach to this coil design did not influence SNR performance when compared to a conventional loop design, these simulations were also performed using the model of the $^{31}P/^{1}H$ endorectal coil in which the microstrip was replaced by a loop coil of equal length and width (Supplemental Figure S-1). This would be an alternative design compact enough to also be installed on top of the plastic rod. This loop coil was not located exactly on top of the rod, but rotated 35° to optimize the orientation of the $B_1^-$ profile. The simulated $^{1}H$ receive profile of the asymmetric coil was validated in phantom measurements (cylinder of diameter 85 mm) with fluid of the same permittivity and conductivity as in the simulations ($\epsilon_r = 43$ and a conductivity of $\sigma =$ 0.72 S/m at a frequency of 297 MHz). Both coils were tuned and matched to $^{1}H$ using fixed capacitors. MR-measurements were performed on a 7 Tesla animal MR system (Clinscan, Bruker Biospin, Ettlingen Germany). A rodent body volume coil was used for transmit and the asymmetric microstrip coil was used to receive the MR signal generated with a gradient echo (GRE) sequence. Simulations of the $^{31}P$ loop coil were performed as well.

$B_1^-$ simulations of the asymmetric $^{1}H$ microstrip showed a similar profile as a loop coil with similar dimensions. The position and rotation of the coil within the balloon, combined with the sensitivity profile, provided a symmetric coil profile and optimized SNR profile with respect to the prostate (Supplemental Figure S-1). Furthermore, the $^{31}P$ $B_1^-$ profile simulations show good overlap with the $B_1^-$ profile of the $^{1}H$ microstrip. There is a minor mismatch at the top of the coil, where the $B_1^-$ profile of the $^{1}H$ microstrip reaches beyond the sensitivity profile of the $^{31}P$ loop coil. The $^{31}P$ loop coil also maintains a higher sensitivity at the bottom of the ERC. As with any endorectal coil, for patients with very large prostates the coverage of the coil might not suffice to cover the complete prostate.

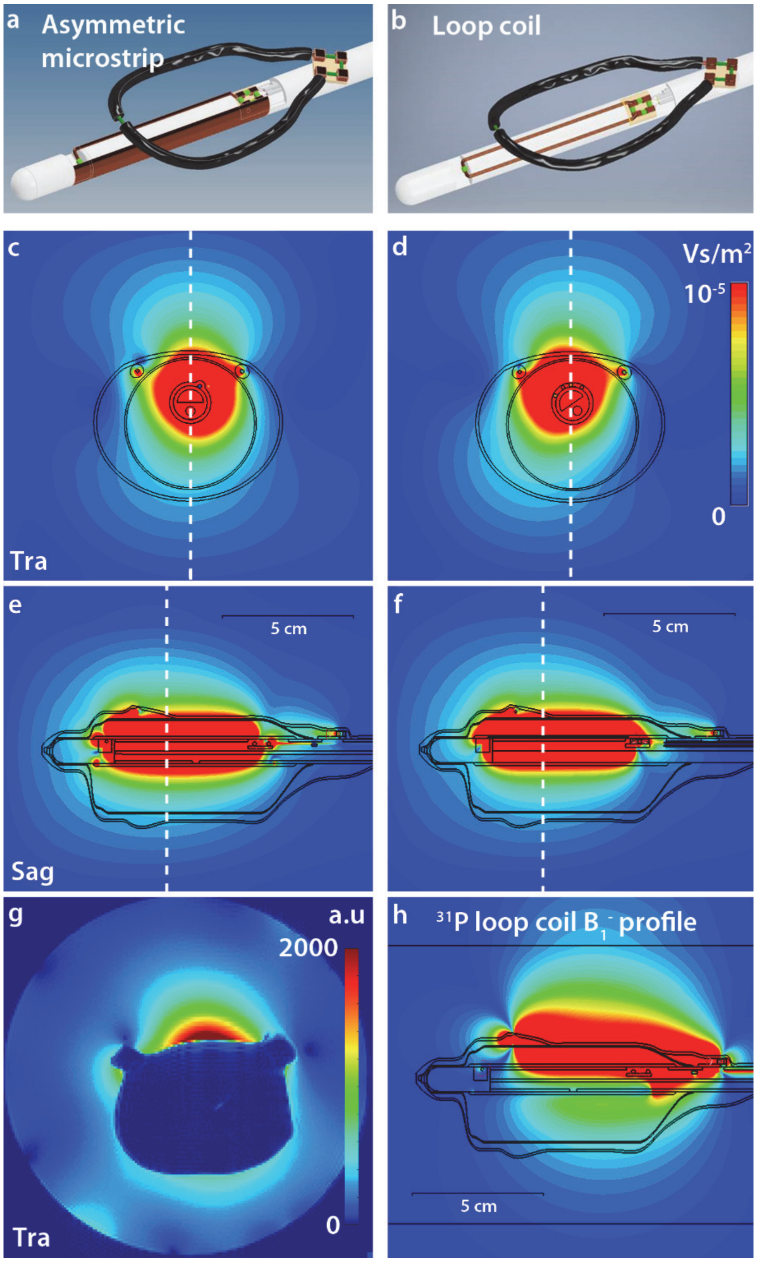


**Supplemental Figure S-1**: $B_1^-$ simulations of the $^1$H Rx asymmetric microstrip (**a,c,e**) and a loop coil with similar length (**b,d,f**). Sagittal (**e,f**) as well as transversal (**c,d**) cross sections of the simulations are shown. The dashed lines in the transversal (sagittal) images indicate the cross section through which the sagittal (transversal) images are shown. The $^1$H simulations are performed with an $\epsilon_r = 43$ and a conductivity of $\sigma = 0.72$ S/m at a frequency of 297 MHz. Simulations of the $B_1^-$ profile through the same sagittal cross section of the $^{31}$P loop coil are shown as well (within fluid with $\epsilon_r = 79$, $\sigma = 0.49$ S/m at a frequency of 120 MHz) (h). A transversal experimentally determined coil profile of the asymmetric microstip is shown in (**g**) and matches the location of the transversal slice in (**c**). The coil was oriented in the same direction as in the simulations.

### S.3 *In vivo* measurement setup and $B_1^+$ with external body array

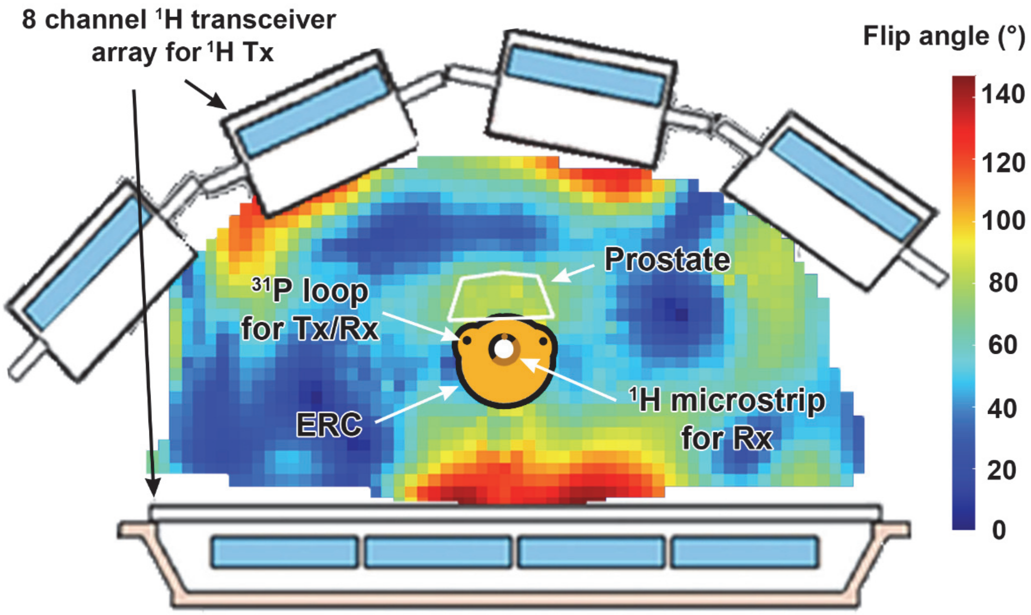


**Supplemental Figure S-2**: A schematic transversal overview of the complete *in vivo* measurement setup, including a transversal flip angle map through the abdomen (volunteer, age 40 years, weight 83 kg) when transmitting with the combined 8-channel coil array after $B_1^+$ shimming. The patient is positioned on top of the bottom part of the 8-channel external body array. The top part of the body array is then placed on top of the patient. After performing the $B_1^+$ shimming sequence, an ROI (indicated by white border) is drawn to indicate the location of the prostate, such that the $B_1^+$ can be optimized for local transmit homogeneity within the prostate. A flip angle distribution of 78.5 ± 3.9 ° was achieved within the ROI, using a block pulse of 1 ms at 300V pulse voltage. The endorectal coil (ERC) is placed endorectally, close to the prostate. The $^{31}$P loop coil for $^{31}$P Tx and Rx and the $^{1}$H microstrip for $^{1}$H Rx are indicated.

## S.4 Additonal patient examples

The seminal vesicles had a high tCho signal on $^{1}H$ spectroscopy, but significantly lower spermine than normal prostate tissue (Supplemental Figure S-3). $^{31}P$ spectroscopy within the vesicles showed an increased ratio of PC/PE compared to the prostate, which is also reflected by the metabolite map. GPC and GPE were undetectable within the seminal vesicles.

It is known that the seminal vesicles and particularly the ejaculatory ducts feature elevated tCho and PC levels and decreased PE signals [2]. The presented case (Supplemental Figure S-3) confirms these results and shows an increased PC/PE ratio and a high tCho signal with an almost absent Spm peak. We did not detect any GPC and GPE in the seminal vesicles, indicating that the elevated tCho signal is in this case not related to any increases in GPE and GPC.

Supplemental Figure S-4 shows an additional patient example.

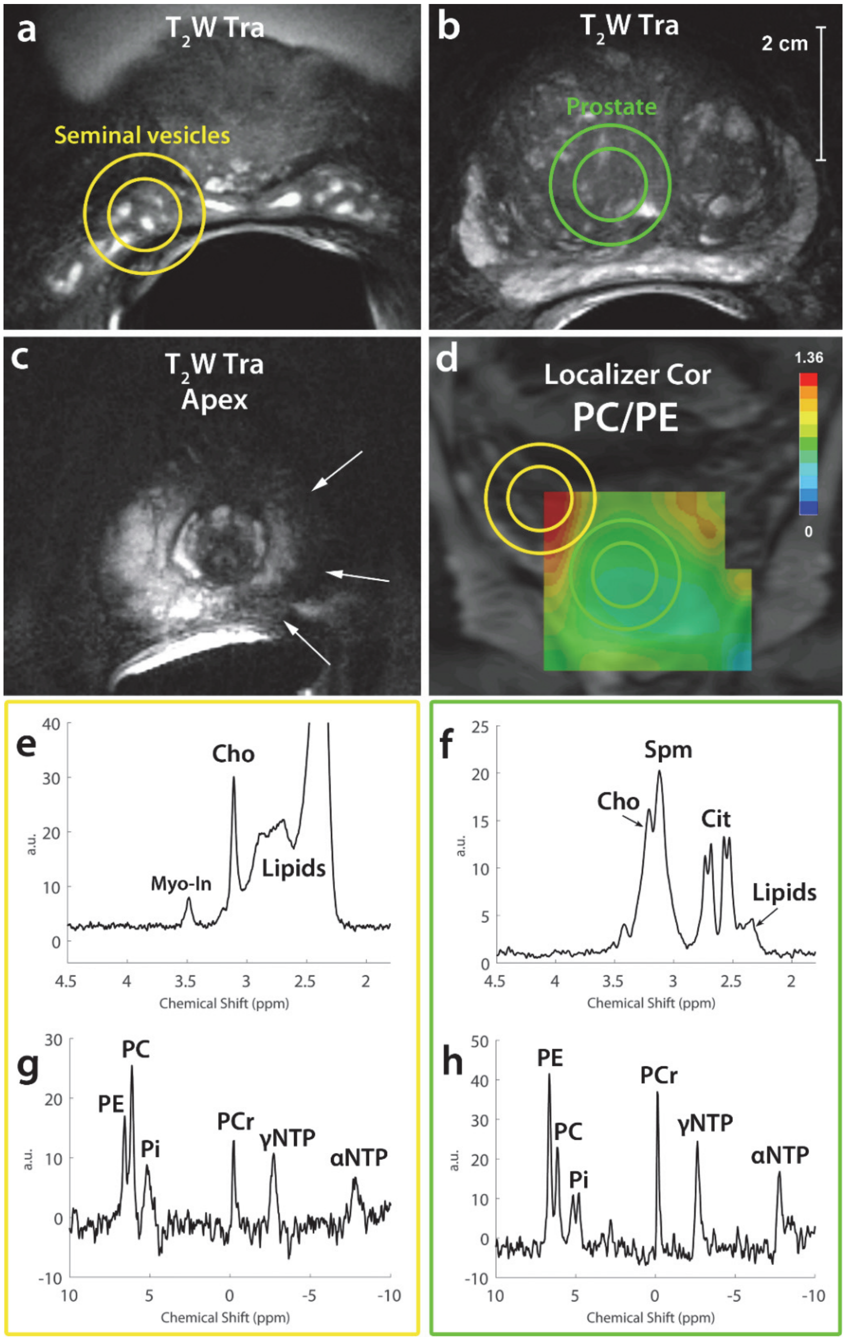


**Supplemental Figure S-3**: Transversal $T_2$W imaging of a patient (60 years old, 97 kg) in the seminal vesicles **(a)**, the mid-prostate **(b)** and the apex **(c)** of the prostate. In addition, a coronal localizer with overlapping metabolite map is shown **(d)**. The metabolite map shows the ratio of PC/PE, which was determined by dividing the integral of the PC peak by the integral of the PE peak. $^{1}$H **(e,f)** and $^{31}$P **(g,h)** spectra are shown of the locations in the correspondingly colored circles (seminal vesicles: yellow; prostate tissue: green). The apex $T_2$W image shows a cancer lesion, annotated by white arrows. The localizer images were obtained with the body array only. $^{31}$P MRSI measurement set b) was used.

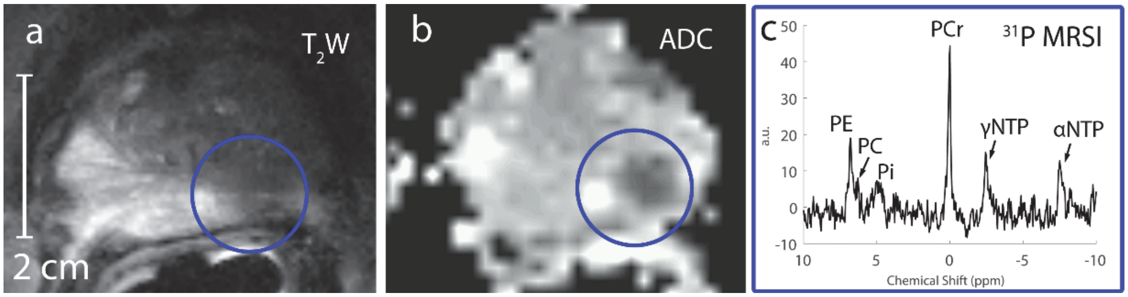


**Supplemental Figure S-4**: ADC map **(a)**, $T_2$W image **(b)** and $^{31}$P MRSI spectrum **(c)** of a patient (63 years, 83 kg) with prostate cancer (Gleason 3+4). The lesion identified as cancer was annotated with a blue circle that also indicates the location of the $^{31}$P spectrum. $^{31}$P MRSI measurement set b) was used.

## References

1. Kobus T, Bitz AK, van Uden MJ, et al. In vivo 31P MR spectroscopic imaging of the human prostate at 7 T: safety and feasibility. *Magn Reson Med.* 2012;68(6):1683-1695.
2. Lagemaat MW, Vos EK, Maas MC, et al. Phosphorus magnetic resonance spectroscopic imaging at 7 T in patients with prostate cancer. *Invest Radiol.* 2014;49(5):363-372.
